# Exploring the inflammatory profile of homelessness population: a comprehensive analysis of individuals in two temporary shelters in Lisbon

**DOI:** 10.3389/fpubh.2024.1432044

**Published:** 2024-09-12

**Authors:** Ana T. P. C. Gomes, Karina Mendes, Cândida Ferrito, Filipa Andrade, João Neves-Amado, Ana Resende, Paulo Santos, Dina Manso, António Almeida, Antónia Vollrath, Rafaela Lopes, Marlene Barros, Nuno Rosa, Amélia Simões Figueiredo

**Affiliations:** ^1^Centre for Interdisciplinary Research in Health (CIIS), Faculty of Dental Medicine, Universidade Católica Portuguesa, Viseu, Portugal; ^2^Centre for Interdisciplinary Research in Health (CIIS), Faculty of Health Science and Nursing, Universidade Católica Portuguesa, Lisbon, Portugal; ^3^Faculty of Health Science and Nursing, Universidade Católica Portuguesa, Lisbon, Portugal; ^4^Faculty of Health Science and Nursing, Universidade Católica Portuguesa, Porto, Portugal; ^5^Núcleo de Planeamento e Intervenção Sem-Abrigo (NPISA), Lisbon, Portugal; ^6^Centre for Interdisciplinary Research in Health, Católica Medical School, Universidade Católica Portuguesa, Lisbon, Portugal; ^7^Facultad de Ciencias para el Cuidado de la Salud, Universidad San Sebastian, Santiago, Chile

**Keywords:** homeless, vulnerable population, nursing, inflammatory profile, healthcare

## Abstract

**Background:**

Homeless people are continuously facing adverse living conditions as poor access to basic nutrition, hygiene conditions and healthcare services, being at increased risk of severe infectious diseases as HIV and hepatitis as well as cardiovascular diseases and mental disorders. The characterization of homeless people’s health is fundamental to identify their health care needs. Considering that the aforementioned diseases are associated with chronic inflammatory processes, the main goal of this study was to characterize the inflammatory profile of a homeless population through quantification in saliva of a panel of inflammatory cytokines.

**Methods:**

The inflammatory profile was assessed in 114 individuals residing in two temporary shelters located in Lisbon and that accepted to participated in the study. Inflammatory proteins were quantified using a Multiplex Immunoassay approach. Data analysis was performed using the GraphPad Prism software and statistical significance among the groups was assessed using the nonparametric Mann–Whitney test.

**Results:**

Even though some protein levels might be masked by drug treatment, data analysis showed high levels of INF-ϒ, IL-10 and TNF-α in the infectious disease group, critical cytokines for the immune response against viruses and bacteria. Also, cytokines like IL-1β and IL-6 were detected at statistically significant levels in the cardiovascular disease group and all cytokines included in this study were quantified in the mental disorders group.

**Conclusion:**

These findings may help the healthcare services in the evaluation of treatment efficacy and disease monitoring, and in the development of effective public healthcare strategies and policy interventions to improve quality of life of the homeless population.

## 1 Introduction

Homelessness is a complex and pressing issue in several parts of the world, highly relevant in the global scenario toward demographic and climate changes, urbanization, and financial crises. In fact, homelessness and housing are topics of concern of the World Health Organization (WHO) (1, 2). Homelessness is rarely the result of a single factor but rather a combination of interconnected issues. Economic instability and unemployment, insufficient affordable housing options, wars that trigger refugee crises and mental health and substances abuse are some of the key challenges that need comprehensive solutions (3, 4). Moreover, the COVID-19 pandemic has exacerbated existing challenges for the homeless population. Issues such as overcrowded shelters, lack of access to hygiene facilities, and disruptions to essential services have made it even more difficult for homeless individuals to protect themselves (5, 6).

In Portugal, the official definition of a person experiencing homelessness (PSSA) was presented in 2009 and is one recommendation of ENIPSSA (according to *Resolução do Conselho de Ministros n° 107/2017 – DR n.° 142/2017, Série I de 25* de julho) and stated: “A person who, regardless of their nationality, age, gender, socioeconomic status or physical and mental health condition, is homelessness, living in a public space, staying in an emergency shelter or in a precarious place; or without a house, staying in temporary accommodation intended for this purpose” (7). Accordantly with Estratégia Nacional Para a Integração de Pessoas em Situação de Sem Abrigo (ENIPSSA), in 2022 more than 10,700 people were in homeless situation, where 3,138 live in the city of Lisbon (2,744 homeless and 394 homelessness). In fact, in the Lisbon metropolitan area there are 1,170 homeless people living in temporary (8). Aiming study the vulnerable populations that attend public bathhouses available in the city of Lisbon, a broad research project emerges - the Public Bathhouse Nursing, integrating research, service delivery, and education. These bathhouses are places where individuals can bathe and obtain clothing. They date back to the 1930s and are municipal resources managed by the city’s Parish Councils, being mostly used by homeless individuals. Some research efforts have been conducted to characterize the bathhouse users’ profile and to identify the respective health needs. Under this project, the bathhouse user profile was found - is a man, single, in the active phase of life, living without any remuneration, with associated mental illness and living in a homeless situation (9, 10). In those cases, it was found that mental illness, social, personal, and family factors justify the transition to homelessness. The total absence of hopelessness alternates with expectations for the future based on resilience and hope, as well as their experiences with respect to health outcomes and parenting under the abovementioned circumstances (11, 12). Nursing, in transdisciplinary work with other disciplinary areas, can respond to the vulnerable population, due to its predominant role in promoting health (11).

Due to the motifs enumerated above it is not difficult to understand that the access to healthcare services of homeless individuals is tough. Overcrowded living conditions, limited access to sanitation, and compromised immune systems, homeless persons are exposed to many communicable infections, such as human immunodeficiency virus (HIV) infection, hepatitis B and C, active tuberculosis, scabies, body lose infestations, etc. (13, 14). Moreover, homelessness is associated with a higher prevalence of chronic health conditions such as cardiovascular disease, diabetes, and respiratory disorders (13, 15, 16). This population often experiences high levels of stress, trauma, and mental health challenges (17, 18).

Thus, it is obvious the need to create and improve the health care management in primary care for homeless people. Knowing the health care needs of this particular population is crucial for understanding and addressing their unique health challenges. Amongst the parameters that could help to characterize homeless people’s health, the inflammatory profile can provide valuable insights. Inflammation is the immune system response triggered by different factors, that is linked to numerous health conditions (19) serving as an essential body’s defense mechanism to prevent infection or disease progression and persistent tissue damage. However, if this inflammatory response is not suppressed it can result in chronic inflammation that may lead to diseases like type 2 diabetes, cardiovascular disease among others.

Inflammatory markers include the pro- and anti-inflammatory cytokines (promote and inhibit inflammation, respectively) that are classified as interleukins (ILs), colony stimulating factors (CSF), interferons (IFNs), tumor necrose factors (TNFs), transformation growth factors (TGFs), and chemokines (19). As suggested in the literature (20), inflammatory cytokines such as IL-1β, IL-6, TNF-α may be used as biomarkers for disease diagnosis, prognosis and therapeutic decision making. Quantification of these inflammatory proteins in saliva has potential applications in different fields, including dentistry and medicine, to diagnose and monitor periodontal disease and systemic inflammatory conditions, respectively (21). Several proteins have been identified in saliva, including cytokines, chemokines, growth factors, enzymes, and other molecules that are involved in the immune response and inflammation (21).

Understanding the inflammatory profile of a population can aid in public health planning and policy development. Identifying prevalent inflammatory conditions within a community can inform targeted interventions, such as vaccination, lifestyle interventions, or public health initiatives to reduce inflammation-related diseases (22, 23). In the context of homeless people, studying inflammation can shed light on the prevalence of infections (24) and on chronic diseases helping healthcare providers monitor and manage the health of those individuals (25). Inflammation has been also linked to mental health disorders. Inflammatory profile can help to monitor disease progression and treatment efficacy in homeless individuals with mental disorders, allowing the development of strategies for mental health support (26, 27).

This work aims to characterize the inflammatory profile of the homeless population that attend two temporary shelters in Lisbon, through quantification of anti- and pro-inflammatory cytokines in saliva samples.

## 2 Materials and methods

### 2.1 Study design and data collection

Homeless individuals who used the Alcântara public bathhouse and/or the Lisbon structures of the Núcleo de Planeamento e Intervenção Sem-Abrigo (NPISA) – an organization that provides support to the homeless population were enrolled in this observational cohort study.

Of the total of 1,170 homeless people living in these temporary accommodations, we recruited 396 residents. Due the nature of our goal, collet data related to systemic health of the participants was imperative, and this was possible only for 84 participants. Nevertheless, a group of 30 participants in which it was not possible to obtain any data on systemic health was included, to represent the participants with no clinical data (no recorded group).

Inclusion criteria comprised individuals resided in two temporary shelters located in Lisbon that accepted participated in the study and gave informed consent for data and saliva collection. The only exclusion criterium was the incapacity to give a written informed consent. The primary work of nursing teams focuses on mental health and involves assisting individuals in overcoming alcohol dependence and other addictive behaviors. These services are primarily accessed by homeless individuals, who seek help from these mental health teams prior to dinner and bedtime. It should be noted that in these temporary shelters there is a rotation of people which makes it difficult to fix the sample and collect more specific data to pursue the investigation. For control purposes, a group of participants who were not homeless and had similar demographic data to the homeless population were also recruited. The inclusion criteria for control group were that they should not have any systemic diseases and should not be on regular medication.

Following the protocol established for health-related information, the coded data was incorporated in the individuals’ clinical records, within the Católica Nursing Centre’s platform. This platform meets the existing ethical and legal requirements for the protection of health-related information and other personal data, being sanctioned by the Portuguese data protection authority (Comissão Nacional de Proteção de Dados) through Proc. No. 9673/2014, and complying with relevant national legislation (“Lei de Informação de Saúde” – Lei n.° 12/2005, de 26 de janeiro).

### 2.2 Saliva sample collection, packaging, and storage

Unstimulated whole saliva samples were collected by spitting, according to previously established procedures (28), and kept refrigerated until transportation. The transportation of the specimens to SalivaTec laboratory complied with the applicable national guidelines (Orientação n° 015/2020 de 23/03/2020, updated on April 24th, 2020), namely as regards the following aspects:

- Immediately after the collection process, the samples were sent to the laboratory as soon as possible, and were kept in a refrigerated environment.
- The specimens were transported by a duly certified company, authorized to carry Category B samples (UN 3373). Since samples have potential biological risk, they were manipulated in a Level II safety laboratory. The UWS samples were aliquoted into several microtubes, depending on the volume initially collected and were immediately stored at −80°C, until further analysis.

### 2.3 Determination of the COVID-19 viral load

Since this study occurred during the COVID-19 pandemic, all participants were screened against COVID-19. Thus, the determination of viral load was performed in pool format and the protocol was adapted from the previously described (28). Briefly, saliva from 10 participants was mixed and treated with QuickExtractTM DNA Extraction Solution lysis buffer (Lucigen) for RNA extraction. The FOSUN commercial kit (Shanghai Fosun Long March Medical Science CO., Ltd., Shanghai, China) was used to determine the viral load by RT-PCR (CFX96TM Real-Time System, BIO-RAD), according to the manufacturer’s instructions. Positive SARS-CoV-2 cases were reported using the National Health Service’s SINAVE platform. To ensure physical isolation, the individuals were referenced to an appropriate support structure (“Estrutura de Apoio de Retaguarda”).

### 2.4 Quantification of inflammatory proteins

Salivary inflammatory proteins were quantified trough Multiplex Immunoassay technology using a commercial Multiplex® Map Human High Sensitivity T Cell Magnetic Bead Panel Kit customized with magnetic microspheres coated with specific antibodies for anti-inflammatory proteins (INF-γ, IL-10 and IL-4) and pro-inflammatory proteins (TNF-α, IL-6 and IL-1β). The selection of this panel of inflammatory proteins were based on the associated diseases of this population, as referred to in the introduction section. Thus, participants saliva samples were centrifuged for 10 min, 10,000 rpm, 4°C and the supernatant was used for Multiplex quantification according to the manufacture and analyzed in a Bio-Plex® MAGPIX™ Multiplex Reader.

### 2.5 Data analysis

Due to the population heterogeneity in terms of medical conditions, participants were grouped into five distinct groups, namely infectious, cardiovascular or multiple diseases, mental disorders and no record. Data were analyzed using the GraphPad Prism 9.0.0 (GraphPad Software, San Diego, CA, USA). The results are presented as the mean of 2 replicates and the respective standard deviation. Statistical significance among the groups was assessed using the nonparametric Mann–Whitney test.

## 3 Results

This study enrolled 396 residents of two temporary accommodation centers. This number was consistent with the pre-established criteria – an acceptable estimation error of 3.6% and a confidence level of 95% (Table 1). Since this study was running during the COVID-19 pandemic, saliva samples of all participants were screened against COVID-19. Of the 396 screened homeless individuals, six tested positive for SARS-CoV-2, and due to the implications that this infection could have on the inflammatory profile, these participants were excluded from this study.

**Table 1:** Sample size determination.

Size of homeless population (Lisbon)	Acceptable estimation error (%)	Estimated sample size	Actual sample size
2,900	3.6	394	396

For the inflammatory profile, collet data related with systemic health was imperative. Only 84 participants have this data recorded and actualized. Nevertheless, a group of 30 participants in which it was not possible to obtain any data on systemic health was included, to represent the participants with no clinical information (no recorded group). Thus, the inflammatory profile was accessed in 114 participants. Table 2 shows the general characterization of this sample, according to the contemplated epidemiological variables.

**Table 2:** Demographic characteristic of the selected participants.

**Gender (*N* = 114)**	Male – 103 (90.3%) Female −10 (8.7%)
Age (*N* = 86)* (Average – 50.9)	19–40 – 18 (20.9%) 41–60 – 44 (51.2%) >60–24 (27.9%)
**Associated diseases** **(*N* = 114)**	Infection diseases – 7 (6.1%) Cardiovascular diseases – 4 (3.5%) Mental disorders – 44 (38.6%) Multiple diseases – 29 (25.4%) No record – 30 (26.3%)

*The exact age of 28 individuals could not be determined.

Regarding to the gender of the participants, the majority were male in a percentage of 90.3%, while female recruited were just 8.7%. The exact age of the participants was only accessed in 86 participants, 20.9% were between 19 and 40 years old, 51.2% with ages between 41 and 60 years old and 24% with age higher than 60 years old. The clinical data about associated diseases of the participants were also collected and were grouped according to the type of disease (Tables 2, 3). Although it was not possible to record the health profile of 30 participants, it was possible to find that 38.6% suffer from mental disorders, most of them being related to alcoholism, drug addiction and schizophrenia. Others suffer from infection diseases (6.1%) related to Tuberculosis, HIV and hepatitis B and C or from cardiovascular diseases (3.5%), mainly related to hypertension. Interestingly, 25.4% of the studied population suffer from multiple diseases that englobe the referred previously, such as mental disorders associated with infection and cardiovascular diseases. In what concerns to the lack of data associated with demographic and medical records, it is important to note that the high rotation of temporally shelters users hamper the data collection.

**Table 3:** Associated diseases of the selected participants.

Associated diseases (*N* = 114)	
**Infectious Diseases (*N* = 7)**	Tuberculosis (*N* = 2, 28.6%); HIV (*N* = 3, 42.8%); Hepatitis B (*N* = 1, 14.3%); Hepatitis C (*N* = 1, 14.3%)
**Cardiovascular Diseases (*N* = 4)**	Heart problems (N = 1, 25%); Hypertension (*N* = 3, 75%)
**Mental disorders (*N* = 44)**	Alcoholism (*N* = 19, 43.2%); Anxiety (*N* = 1, 2.3%); Drug Addition (*N* = 6, 13.6%); Epilepsy (*N* = 3, 6.8%); Schizophrenia (*N* = 6, 13.6%); Border line syndrome (*N* = 1, 2.3%); Psychiatric illness not diagnosed (*N* = 2, 4.54%); Depression (*N* = 5, 11.4%); Bipolar (*N* = 1, 2.3%).
**Multiple diseases (*N* = 29)**	Infection diseases + cardiovascular diseases + Mental disorders
**No record (*N* = 30)**	With data available

The inflammatory profile of the 114 participants was determined by quantifying three anti-inflammatory cytokines (INF-γ, IL-10 and IL-4) and three pro-inflammatory cytokines (IL-1β, IL-6 and TNF-α), using xMAP® multiplex assays. The IL-4 results were excluded, since the quantification of this interleukin was below the method’s detection threshold in all the samples evaluated.

**Figure 1:**
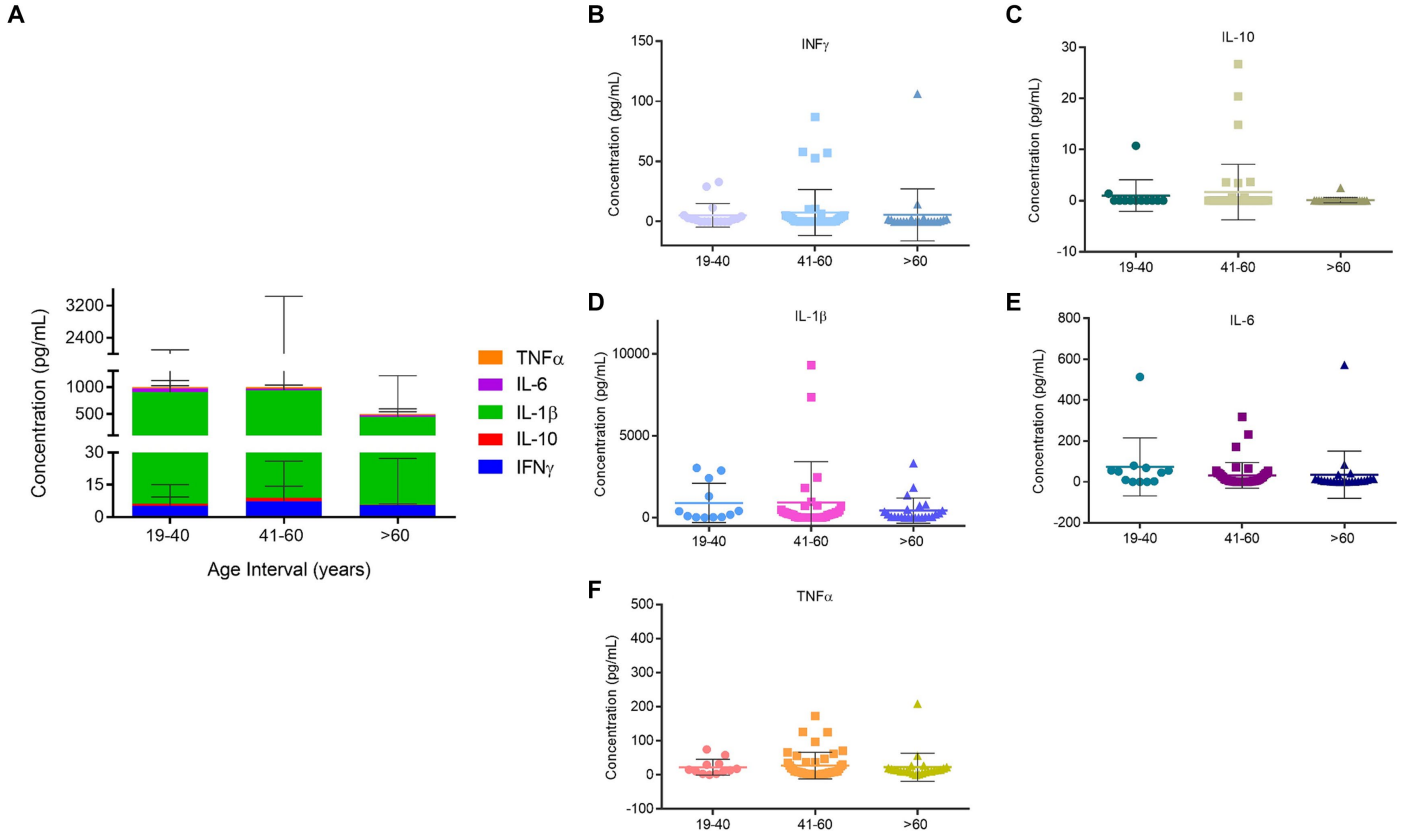
Quantification of inflammatory proteins of the homeless population stratified by age. **(A)** Inflammatory proteins concentrations amongst age groups. **(B)** INF-ϒ concentration in the different age groups. **(C)** IL-10 concentration in the different age groups. **(D)** IL-1β concentration in the different age groups. **(E)** IL-6 concentration in the different age groups. **(F)** TNF-α concentration in the different age groups. Data are the mean value ± SD of at least two duplicates.

Knowing that there is a strong association between the inflammatory profile and the process of aging, the first attempt to characterize this population was based on the evaluation of the age effect in the protein inflammatory quantification. The results presented in Figure 1 show that no significant differences were observed amongst the different age groups (Figure 1A), however it is possible to note that in all cases, IL-1β is present in higher concentrations when compared with the other interleukins (Figure 1D).

**Figure 2:**
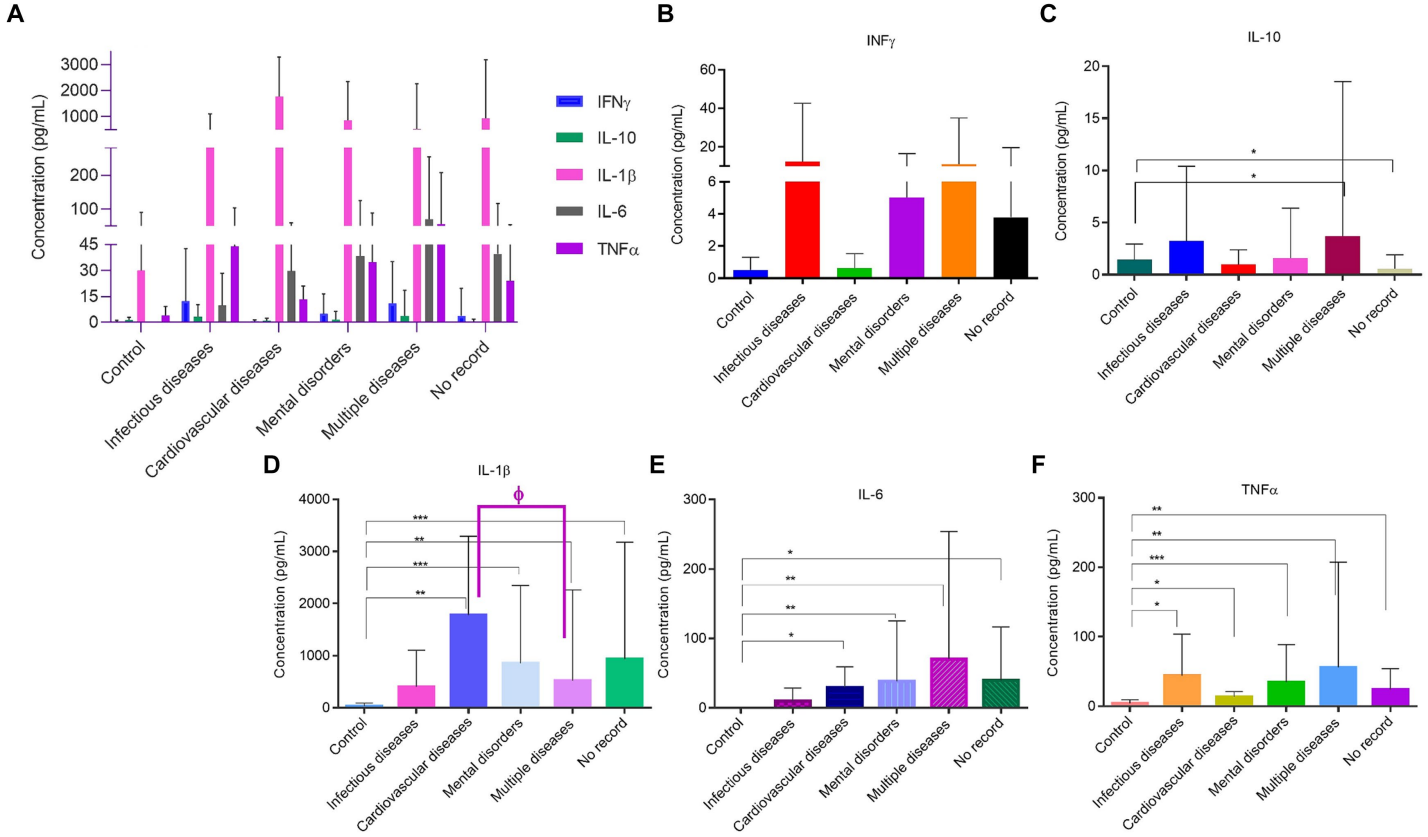
Quantification of inflammatory proteins of the homeless population stratified by associated diseases. **(A)** Inflammatory proteins concentrations amongst associated diseases groups. **(B)** INF-ϒ concentration in the different associated diseases groups. **(C)** IL-10 concentration in the different associated diseases groups. **(D)** IL-1β concentration in the different associated diseases groups. **(E)** IL-6 concentration in the different associated diseases groups. **(F)** TNF-α concentration in the different associated diseases groups. Data are the mean value ± SD of at least two duplicates. *(*p* < 0.01); **(*p* < 0.05); ***(*p* < 0.001) compared to Control. ɸ (p < 0.01) comparing cardiovascular with multiple diseases.

The selection process for participants in the control group considered the demographic traits of the homeless population under study. Additionally, none of the individuals in the control group had any systemic diseases or and were on regular medication (Table 4). The results achieved for the inflammatory profile are presented in Figure 2 and show that for INF-ϒ not significant difference from control were attained (Figure 2A). However, in the remaining proteins a different profile was observed. IL-10 appeared increased in participants with multiple diseases (*p* < 0.01) and decreased in the No Record group (p < 0.01) (Figure 2C). Also in this case, IL-1β appears increased in all groups when compared to the control (Figure 2D). The major increase in this interleukin was observed in the cardiovascular diseases group with a concentration of 1778 ± 1,512 pg./mL (*p* < 0.05), followed by No Record group with 934.1 ± 2,245 pg./mL (*p* < 0.001), Mental disorders with 855.5 ± 1,493 pg./mL (p < 0.001) and multiple diseases group with 522 ± 1736 pg./mL (*p* < 0.05) (Figure 2D). It is worth mentioning that only in the quantification of this interleukin was possible to find statistical significance amongst the associated diseases groups, namely between the cardiovascular and multiple diseases (*p* < 0.01). IL-6 was shown to be increased in all associated diseases, except for the infection diseases group (Figure 2E). For this protein, multiple diseases group registered the highest value with a concentration of 70.5 ± 183.6 pg./mL (*p* < 0.05) (Figure 2E). A similar profile was achieved for TNF-α, where this inflammatory protein was increased in all associated diseases when compared to control (Figure 2F). It is important to highlight the results from Infection and multiple diseases groups, due to the higher concentrations achieved [43.9 ± 59.9 pg./mL (*p* < 0.01) and 55.8 ± 151.6 pg./mL (*p* < 0.05), respectively] (Figure 2F).

**Table 4:** Demographic characteristic of the participants in the control group.

**Gender (*N* = 9)**	Male – 5 (55.6%) Female −4 (44.4%)
**Age (*N* = 9)** (Average – 46.4)	19–40 – 2 (22.2%) 41–60 – 6 (66.6%) >60–1 (11.1%)

## 4 Discussion

Serious health problems, such as Human Immunodeficiency Virus/Acquired Immunodeficiency Syndrome (HIV/AIDS), Hepatitis A infections, alcohol and drug addiction and mental illness are prevalent in homeless populations as supported by our study (29). Consequently, the homeless population suffering with these clinical conditions exhibits impairment of immune function which might be associated to the higher morbidity and mortality observed in this population. Also, the malnutrition issues that homeless people face daily may impair immune responses and thus, the body’s ability to fight a certain infection or disease (30).

It has been reported that older population is more vulnerable to infections, and chronic conditions, such as cardiovascular disease, diabetes, physical disability, and cognitive decline, resulting in increased mortality, and morbidity (31, 32). Inflammation has been described as an endogenous factor in aging, with IL-6, TNF-α and C-reactive protein (CRP) being associated with age-related chronic diseases and disability (31). Considering that, around 27% of the population in this study is older than 60 years old and have comorbidities like mental disorders it would be expected a higher and statistically significant level of inflammatory cytokines mainly IL-6 and TNF-α in these individuals compared to the younger age group. However, a correlation between aging and inflammatory profile was not observed in this population, which might be explained by the high prevalence of comorbidities in these people as indicated by the high levels of IL-1β across all age groups.

Besides the association between aging and inflammation, sex differences in immune response have been reported as well (33). Although it would be interesting to correlate the inflammatory marker levels between females and males, the unequal number of individuals of each sex, 103 males and only 10 females, makes this analysis unfeasible. Most diagnostic and characterization studies of homeless people report gender disparities, with greater expression in males (34). In Portugal, 68% of homeless people are male (8). Although the exact reasons remain uncertain, some studies propose that the responsibility of caring for their children may provide homeless women with alternative support systems, potentially leading them away from life on the streets (35, 36).

The inflammatory profile of this homeless population was correlated with the different clinical conditions of these participants. When looking at the anti-inflammatory cytokine profile, participants with infectious diseases showed high levels of INF-ϒ and IL-10. Indeed, it has been reported for both cytokines a critical immunoregulator role during infection with viruses, bacteria, fungi, protozoa and helminths, thus conferring protective immunity in infectious diseases (37, 38). For instance, INF-ϒ and IL-10 are involved in HIV pathogenesis with production of INF-ϒ and IL-10 across the course of infection. In a similar way anti-retroviral therapy was shown to decrease the IL-10 levels in serum (39). Considering that some participants from this study were under anti-retroviral therapy for HIV, it’s expected that IL-10 and other cytokines levels quantified in saliva samples might be masked. Still focusing on infectious disease, high levels of TNF-α were detected in participants from this group, which is in line with the role of this pro-inflammatory cytokine in the pathogenesis of immune-mediated diseases such as HIV and tuberculosis (40, 41).

While most of the cytokines quantified in this study displayed increasing levels in the infectious disease group compared to the remaining groups, IL-6, a pro-inflammatory cytokine, was the exception and showed decreasing levels. Despite the IL-6 role in diseases related to virus infection like hepatitis and HIV, the lower levels detected in the infectious diseases group might be explained by some participants undergoing anti-retroviral therapy (42).

As already mentioned, mental disorders have high prevalence rates in homeless people (43). Indeed, 44 from 114 participants included in this study had a mental health disorder. Cytokines have been reported to be critical for the normal development and functioning of brain activity. However, chronic production and high levels of inflammatory cytokines may be associated with development of neuropsychiatric disorders and depression in patients with or without additional chronic medical ill-nesses (44). Also, patients with mental disorders like major depression have a greater risk of comorbidity and mortality (44). In fact, a substantial number of participants (29 from 114) within the multiple diseases group presented two or more chronic diseases including mental disorders, which may explain the statistically significant cytokine levels observed in this group compared to the control group. This data shows that innate immune activation in participants with more than one chronic medical condition have higher levels of inflammatory cytokines. Once again, the cytokine levels detected in all disease groups might be masked by drug treatment such as antidepressant and anti-retroviral therapy, among others. Taking into consideration that some patients are not responsive to antidepressant treatment and thus have higher concentrations of inflammatory cytokines than patients who respond to the treatment (44), the evaluation of the inflammatory profile in the homeless population with these medical illnesses may help the healthcare services in the management of treatment efficacy on this population, implementing new strategies to improve the health of homeless people.

It is well known the role of inflammation in chronic diseases such as cardiovascular diseases with cytokines exerting an important function in cardiac inflammation. Cytokines like IL-1β, IL-6, TNF-α, and IFN-ϒ have been associated with this cardiac inflammation, while IL-10 has been shown to reduce cardiovascular inflammation (45). Despite the reduced number of participants in the cardiovascular disease group (only 4 participants), all those inflammatory cytokines were detected in this disease group, with statistically significant levels (compared to control group) being observed mainly for IL-1β and IL-6. These findings are corroborated by literature, indicating that elevated levels of IL-1β released from immune cells, injured endothelial cells and cardiac cells augments cardiac inflammation. In a similar way, IL-6, one of the most studied cytokines in cardiovascular inflammation, is increased in cardiovascular diseases (45).

Although the higher levels of IL-1β were observed mainly for the cardiovascular disease group, the concentration of this cytokine compared to the other cytokines was extremely elevated in all disease groups, including the no record group. This is in line with IL-1β function as a critical mediator of the immune response in multiple acute and chronic inflammatory diseases (46). Considering that inflammatory cytokines such as IL-1β are also elevated in oral chronic inflammatory diseases like periodontitis (47) and that the homeless people have poor oral health and limited opportunities to dental care (48) cytokines concentration may be affected by undiagnosed oral diseases. In addition, patients with periodontitis have a higher risk for cardiovascular diseases (49) which could be associated with the elevated levels of IL-1β detected in the cardiovascular disease group. This data reinforces the need for oral health interventions for homeless people to improve their dental care and quality of life.

Overall, the results from this study should be interpreted carefully because the number of participants in each group is low and not uniformly distributed and bias in results may occur. Thus, in future experiments the number of participants should be more balanced, mainly for the cardiovascular and infectious diseases groups as well as the control group that need to be matched in age and gender. Another limitation of this study was the difficulty in obtaining medical and drug treatment records for all home-less people as happened with the participants in the no record group. Despite the lack of clinical information in the no record group the results may provide important clues about the health status of these participants. For instance, the elevated levels of pro-inflammatory cytokines when compared to control group indicate that these participants may undergo an acute or chronic inflammatory process. In such cases, participants can be referred to the public healthcare services to assess, treat and follow up the eventual clinical condition.

In the future would be interesting to characterize a higher number of homeless people and expand the inflammatory cytokine panel, quantifying other molecules that have been reported in chronic diseases that are prevalent in the homeless population, such as cardiovascular (IL-38, IL-18, α-amilase) (49) infectious diseases (IL-12 and TGF-α) (19) and mental disorders (IL-18, C- reactive protein) (26). Data from this study will return to the nurses in these public shelters in order to help in the management of global health conditions of homeless people and also to support the development of a network with the public healthcare and policy systems to assist these individuals, improving their quality of life.

## 5 Conclusion

Considering the high prevalence rate of chronic inflammatory diseases in homeless people, it is relevant to characterize the inflammatory profile of these individuals to offer additional tools to the healthcare services in the evaluation of clinical aspects such as treatment efficacy and disease and/or global health monitoring.

The results from this study provide important clues about the health status of the homeless population through quantification of the most relevant inflammatory cytokines associated with chronic inflammatory diseases. It also has the potential to infer about treatment efficacy of those people that undergo drug treatment, and whose inflammatory profile remains unchanged. Since cytokine quantification is performed in saliva, a non-invasive and painless method to collect biologic samples, individuals may be more open to integrate these types of studies. Homeless participants from this and other similar studies may be referred to the public healthcare services to diagnosis, treat and manage their health problems in cooperation with specific policy entities.

## Data availability statement

The original contributions presented in the study are included in the article/supplementary material, further inquiries can be directed to the corresponding authors.

## Ethics statement

The studies involving humans were approved by the Comissão de Ética para a Saúde da Universidade Católica Portuguesa. The studies were conducted in accordance with the local legislation and institutional requirements. The participants provided their written informed consent to participate in this study.

## Author contributions

AG: Data curation, Formal analysis, Investigation, Methodology, Writing – original draft, Writing – review & editing. KM: Data curation, Formal analysis, Investigation, Methodology, Writing – original draft, Writing – review & editing. CF: Methodology, Writing – review & editing. FA: Methodology, Writing – review & editing. JN-A: Methodology, Writing – review & editing. AR: Methodology, Writing – review & editing. PS: Methodology, Writing – review & editing. DM: Methodology, Writing – review & editing. AA: Writing – review & editing. AV: Writing – review & editing. RL: Methodology, Writing – review & editing. MB: Validation, Writing – review & editing. NR: Conceptualization, Data curation, Formal analysis, Investigation, Methodology, Resources, Writing – original draft, Writing – review & editing. AF: Conceptualization, Investigation, Project administration, Resources, Supervision, Validation, Writing – original draft, Writing – review & editing.

## References

[1] Omerov P Craftman ÅG Mattsson E Klarare A. Homeless persons’ experiences of health- and social care: a systematic integrative review. Health Soc Care Community. (2020) 28:1–11. doi: 10.1111/hsc.12857, PMID: 31524327

[2] Available at: https://www.who.int/publications/i/item/9789241550376 (Accessed February 2024).

[3] Mago VK Morden HK Fritz C Wu T Namazi S Geranmayeh P. Analyzing the impact of social factors on homelessness: a fuzzy cognitive map approach. BMC Med Inform Decis Mak. (2013) 13:1–19. doi: 10.1186/1472-6947-13-94, PMID: 23971944 PMC3766254

[4] Available at: https://ec.europa.eu/commission/presscorner/detail/en/ip_21_3044 (Accessed February 2024).

[5] Perri M Dosani N Hwang SW. COVID-19 and people experiencing homelessness: challenges and mitigation strategies. CMAJ. (2020) 192:E716–9. doi: 10.1503/cmaj.200834, PMID: 32601252 PMC7828890

[6] Rodriguez NM Lahey AM MacNeill JJ Martinez RG Teo NE Ruiz Y. Homelessness during COVID-19: challenges, responses, and lessons learned from homeless service providers in Tippecanoe County, Indiana. BMC Public Health. (2021) 21:1657. doi: 10.1186/s12889-021-11687-8, PMID: 34507565 PMC8432956

[7] Available at: https://diariodarepublica.pt/dr/detalhe/resolucao-conselho-ministros/107-2017-107745746.

[8] Available at: https://www.enipssa.pt/-/resultados-do-inquerito-de-caracterizacao-das-pessoas-em-situacao-de-sem-abrigo-dez-20-2 (Accessed February 2024).

[9] Figueiredo AS Resende A Ferrito C Rabiais I Caldeira S. Users of the public bathhouse of Alcântara: health profile diagnosis. Revista de Enfermagem Referencia. (2016) IV Série:107–14. doi: 10.12707/RIV16001

[10] Simões Figueiredo A Vidal T Sarreira-Santos A Medeiros-Garcia L García-Padilla F Seabra P. Nursing consultation in public showers: what lies beyond the results? Issues Ment Health Nurs. (2019) 40:535–6. doi: 10.1080/01612840.2019.1587654, PMID: 30985237

[11] Andrade FMR Figueiredo AS Capelas ML Charepe Z Deodato S. Experiences of homeless families in parenthood: a systematic review and synthesis of qualitative evidence. Int J Environ Res Public Health. (2020) 17:535–536. doi: 10.3390/ijerph17082712, PMID: 32326513 PMC7215402

[12] Figueiredo AS Ferrito C Santos AS Deodato S Seabra P Vidal T. Family transitions to homelessness: a qualitative approach. Rev Bras Enferm. (2020) 73:e20190554. doi: 10.1590/0034-7167-2019-0554, PMID: 32667407

[13] Badiaga S Raoult D Brouqui P. Preventing and controlling emerging and reemerging transmissible diseases in the homeless. Emerg Infect Dis. (2008) 14:1353–9. doi: 10.3201/eid1409.080204, PMID: 18760000 PMC2603102

[14] Noland DH Morris CD Kayser AM Garver-Apgar CE. Results of a peer navigator program to address chronic illness among persons experiencing homelessness. J Community Health. (2023) 48:606–15. doi: 10.1007/s10900-023-01194-9, PMID: 36802004

[15] Zhu A Bruketa E Svoboda T Patel J Elmi N El-Khechen Richandi G. Respiratory infectious disease outbreaks among people experiencing homelessness: a systematic review of prevention and mitigation strategies. Ann Epidemiol. (2023) 77:127–35. doi: 10.1016/J.ANNEPIDEM.2022.03.004, PMID: 35342013

[16] Liu CY Chai SJ Watt JP. Communicable disease among people experiencing homelessness in California. Epidemiol Infect. (2020) 148:e85. doi: 10.1017/S0950268820000722, PMID: 32223777 PMC7189346

[17] Schiffler T Kapan A Gansterer A Pass T Lehner L Gil-Salmeron A. Characteristics and effectiveness of co-designed mental health interventions in primary Care for People Experiencing Homelessness: a systematic review. Int J Environ Res Public Health. (2023) 20:127–135. doi: 10.3390/ijerph20010892, PMID: 36613214 PMC9820061

[18] Zhao E. The key factors contributing to the persistence of homelessness. Int J Sust Dev World. (2023) 30:1–5. doi: 10.1080/13504509.2022.2120109

[19] Chen L Deng H Cui H Fang J Zuo Z Deng J. Inflammatory responses and inflammation-associated diseases in organs. Oncotarget. (2018) 9:7204–18. doi: 10.18632/oncotarget.23208, PMID: 29467962 PMC5805548

[20] Gupta J Mitra N Kanetsky PA Devaney J Wing MR Reilly M. Association between albuminuria, kidney function, and inflammatory biomarker profile in CKD in CRIC. Clin J Am Soc Nephrol. (2012) 7:1938–46. doi: 10.2215/CJN.03500412, PMID: 23024164 PMC3513744

[21] Song M Bai H Zhang P Zhou X Ying B. Promising applications of human-derived saliva biomarker testing in clinical diagnostics. *Int*. J Oral Sci. (2023) 15:1–17. doi: 10.1038/s41368-022-00209-w PMC9810734 36596771

[22] Soehnlein O Libby P. Targeting inflammation in atherosclerosis — from experimental insights to the clinic. Nat Rev Drug Discov. (2021) 20:589–610. doi: 10.1038/s41573-021-00198-1, PMID: 33976384 PMC8112476

[23] Margină D Ungurianu A Purdel C Tsoukalas D Sarandi E Thanasoula M. Chronic inflammation in the context of everyday life: dietary changes as mitigating factors. Int J Environ Res Public Health. (2020) 17:1–27. doi: 10.3390/ijerph17114135, PMID: 32531935 PMC7312944

[24] Chakravarty S Chakravarti R Chattopadhyay S. Inflammatory control of viral infection. Viruses. (2023) 15:1–5. doi: 10.3390/v15071579, PMID: 37515265 PMC10383133

[25] Furman D Campisi J Verdin E Carrera-Bastos P Targ S Franceschi C. Chronic inflammation in the etiology of disease across the life span. Nat Med. (2019) 25:1822–32. doi: 10.1038/s41591-019-0675-0, PMID: 31806905 PMC7147972

[26] Yuan N Chen Y Xia Y Dai J Liu C. Inflammation-related biomarkers in major psychiatric disorders: a cross-disorder assessment of reproducibility and specificity in 43 meta-analyses. Transl Psychiatry. (2019) 9:1–13. doi: 10.1038/s41398-019-0570-y 31534116 PMC6751188

[27] Ouabbou S He Y Butler K Tsuang M. Inflammation in mental disorders: is the microbiota the missing link? Neurosci Bull. (2020) 36:1071–84. doi: 10.1007/s12264-020-00535-1, PMID: 32592144 PMC7475155

[28] Esteves E Mendes AK Barros M Figueiredo C Andrade J Capelo J. Population wide testing pooling strategy for SARS-CoV-2 detection using saliva. PLoS One. (2022) 17:e0263033. doi: 10.1371/journal.pone.0263033, PMID: 35089942 PMC8797214

[29] Sleet DA Francescutti LH. Homelessness and public health: a focus on strategies and solutions. Int J Environ Res Public Health. (2021) 18:1–16. doi: 10.3390/ijerph182111660, PMID: 34770173 PMC8583397

[30] Arranz L De Vicente A Muñoz M De La Fuente M. Impaired immune function in a homeless population with stress-related disorders. Neuroimmunomodulation. (2009) 16:251–60. doi: 10.1159/000212386 19365149

[31] Li X Li C Zhang W Wang Y Qian P Huang H. Inflammation and aging: Signaling pathways and intervention therapies. Signal Transduct Target Ther. (2023) 8:239. doi: 10.1038/s41392-023-01502-8, PMID: 37291105 PMC10248351

[32] Singh T Newman AB. Inflammatory markers in population studies of aging. Ageing Res Rev. (2011) 10:319–29. doi: 10.1016/J.ARR.2010.11.002, PMID: 21145432 PMC3098911

[33] Klein SL Flanagan KL. Sex differences in immune responses. Nat Rev Immunol. (2016) 16:626–38. doi: 10.1038/nri.2016.90 27546235

[34] DiGuiseppi G Semborski S Rhoades H Goldbach J Henwood BF. Perceived safety in community and service settings among young adults experiencing homelessness: differences by sexual and gender identity. Am J Community Psychol. (2022) 70:340–51. doi: 10.1002/ajcp.12606, PMID: 35707878 PMC10083956

[35] Forchuk C Russell G Richardson J Perreault C Hassan H Lucyk B. Family matters in Canada: understanding and addressing family homelessness in Ontario. BMC Public Health. (2022) 22:614. doi: 10.1186/s12889-022-13028-9, PMID: 35351039 PMC8966253

[36] https://www.focusireland.ie/wp-content/uploads/2021/12/Domestic-Violence-and-Family-Homelessness-Report_FINAL.pdf (Accessed February 2024).

[37] Kak G Raza M Tiwari BK. Interferon-gamma (IFN-γ): exploring its implications in infectious diseases. Biomol Concepts. (2018) 9:64–79. doi: 10.1515/bmc-2018-0007, PMID: 29856726

[38] Couper KN Blount DG Riley EM. IL-10: the master regulator of immunity to infection. J Immunol. (2008) 180:5771–7. doi: 10.4049/jimmunol.180.9.5771 18424693

[39] Stylianou E Aukrust P Kvale D Müller F FrØland SS. IL-10 in HIV infection: increasing serum IL-10 levels with disease progression-down-regulatory effect of potent anti-retroviral therapy. Clin Exp Immunol. (1999) 116:115–20. doi: 10.1046/j.1365-2249.1999.00865.x, PMID: 10209514 PMC1905221

[40] Kumar A Coquard L Herbein G. Targeting TNF-alpha in HIV-1 infection. Curr Drug Targets. (2015) 17:15–22. doi: 10.2174/1573399811666150615145824 26073859

[41] Dorhoi A Kaufmann SHE. Tumor necrosis factor alpha in mycobacterial infection. Semin Immunol. (2014) 26:203–9. doi: 10.1016/J.SMIM.2014.04.003 24819298

[42] Li YS Ren HC Cao JH. Roles of Interleukin-6-mediated immunometabolic reprogramming in COVID-19 and other viral infection-associated diseases. Int Immunopharmacol. (2022) 110:109005. doi: 10.1016/J.INTIMP.2022.109005, PMID: 35780641 PMC9236983

[43] Gutwinski S Schreiter S Deutscher K Fazel S. The prevalence of mental disorders among homeless people in high-income countries: an updated systematic review and metaregression analysis. PLoS Med. (2021) 18:e1003750. doi: 10.1371/journal.pmed.1003750, PMID: 34424908 PMC8423293

[44] Felger JC Lotrich FE. Inflammatory cytokines in depression: neurobiological mechanisms and therapeutic implications. Neuroscience. (2013) 246:199–229. doi: 10.1016/j.neuroscience.2013.04.060, PMID: 23644052 PMC3741070

[45] Goswami SK Ranjan P Dutta RK Verma SK. Management of inflammation in cardiovascular diseases. Pharmacol Res. (2021) 173:105912. doi: 10.1016/j.phrs.2021.105912, PMID: 34562603 PMC8541927

[46] Lopez-Castejon G Brough D. Understanding the mechanism of IL-1β secretion. Cytokine Growth Factor Rev. (2011) 22:189–95. doi: 10.1016/j.cytogfr.2011.10.001 22019906 PMC3714593

[47] Cheng R Wu Z Li M Shao M Hu T. Interleukin-1β is a potential therapeutic target for periodontitis: a narrative review. Int J Oral Sci. (2020) 12:2. doi: 10.1038/s41368-019-0068-8, PMID: 31900383 PMC6949296

[48] Freitas DJ Kaplan LM Tieu L Ponath C Guzman D Kushel M. Oral health and access to dental care among older homeless adults: results from the HOPE HOME study. J Public Health Dent. (2019) 79:3–9. doi: 10.1111/jphd.12288, PMID: 30295922 PMC6420347

[49] Sanz M Marco del Castillo A Jepsen S Gonzalez-Juanatey JR D’Aiuto F Bouchard P. Periodontitis and cardiovascular diseases: consensus report. J Clin Periodontol. (2020) 47:268–88. doi: 10.1111/jcpe.13189, PMID: 32011025 PMC7027895

